# Accurate Depth of Radiofrequency-Induced Lesions in Renal Sympathetic Denervation Based on a Fine Histological Sectioning Approach in a Porcine Model

**DOI:** 10.1161/CIRCINTERVENTIONS.117.005779

**Published:** 2018-02-13

**Authors:** Atsushi Sakaoka, Hisako Terao, Shintaro Nakamura, Hitomi Hagiwara, Toshihito Furukawa, Kiyoshi Matsumura, Kenichi Sakakura

**Affiliations:** From Evaluation Center, R&D Administration and Promotion Department, Terumo Corporation, Kanagawa, Japan (A.S., H.T., S.N., H.H.); Graduate School of Engineering, Osaka Institute of Technology, Japan (A.S., K.M.); Biostatistical Research Co, Ltd, Tokyo, Japan (T.F.); and Division of Cardiovascular Medicine, Saitama Medical Center, Jichi Medical University, Saitama, Japan (K.S.).

**Keywords:** denervation, electric impedance, electrodes, pathology, renal artery

## Abstract

**Background—:**

Ablation lesion depth caused by radiofrequency-based renal denervation (RDN) was limited to <4 mm in previous animal studies, suggesting that radiofrequency-RDN cannot ablate a substantial percentage of renal sympathetic nerves. We aimed to define the true lesion depth achieved with radiofrequency-RDN using a fine sectioning method and to investigate biophysical parameters that could predict lesion depth.

**Methods and Results—:**

Radiofrequency was delivered to 87 sites in 14 renal arteries from 9 farm pigs at various ablation settings: 2, 4, 6, and 9 W for 60 seconds and 6 W for 120 seconds. Electric impedance and electrode temperature were recorded during ablation. At 7 days, 2470 histological sections were obtained from the treated arteries. Maximum lesion depth increased at 2 to 6 W, peaking at 6.53 (95% confidence interval, 4.27–8.78) mm under the 6 W/60 s condition. It was not augmented by greater power (9 W) or longer duration (120 seconds). There were statistically significant tendencies at 6 and 9 W, with higher injury scores in the media, nerves, arterioles, and fat. Maximum lesion depth was positively correlated with impedance reduction and peak electrode temperature (Pearson correlation coefficients were 0.59 and 0.53, respectively).

**Conclusions—:**

Lesion depth was 6.5 mm for radiofrequency-RDN at 6 W/60 s. The impedance reduction and peak electrode temperature during ablation were closely associated with lesion depth. Hence, these biophysical parameters could provide prompt feedback during radiofrequency-RDN procedures in the clinical setting.

**WHAT IS KNOWN**

- Although radiofrequency renal sympathetic denervation (RF-RDN) has been proposed as a new treatment option for patients with hypertension, its clinical efficacy has not been established, partly because of the failure in the SYMPLICITY HTN-3 trial (Renal Denervation in Patients With Uncontrolled Hypertension).
- Previous animal studies indicated that the average lesion depth in RF-RDN was 3 to 4 mm, suggesting that RF-RDN might not denervate a substantial percentage of nerves surrounding the human renal artery.

**WHAT THE STUDY ADDS**

- A fine sectioning method (500-μm intervals) revealed that the true lesion depth in RF-RDN was >6 mm, which was deeper than the previously reported depth. A 6-mm depth would cover most of the renal sympathetic nerves in humans.
- Because further augmentation of the lesion depth was not found at a greater output power (9 W/60 s) or longer duration (6 W/120 s), 6 W/60 s was the optimal output power and duration for the multielectrode RF-RDN device.
- Biophysical parameters such as impedance reduction and peak electrode temperature were closely associated with the lesion depth, indicating that these parameters provide prompt feedback during RF-RDN in the clinical setting.

Because the hyperactivity of renal sympathetic nerves plays a key role in the pathophysiological mechanism of hypertension,^1^ catheter-based renal sympathetic denervation (RDN) has been developed as a treatment option for hypertension. Radiofrequency-RDN is still most frequently used in the clinical setting.^2^ Although the safety of radiofrequency-RDN has been confirmed in clinical trials,^3,4^ its efficacy has not yet been established, partly because the SYMPLICITY HTN-3 trial (Renal Denervation in Patients With Uncontrolled Hypertension) could not prove its efficacy.^3^ One possible explanation for the results of the SYMPLICITY HTN-3 trial is the existence of human renal nerves beyond the reach of radiofrequency energy. A recent human anatomic study revealed that 41% and 28% of renal sympathetic nerves are distributed deeper than 3 and 4 mm, respectively, from the renal arterial lumen.^5^ Because previous animal studies indicated that the average lesion depth achieved with radiofrequency-RDN was 3 to 4 mm,^6–8^ radiofrequency-RDN might denervate only a negligible percentage of nerves surrounding the human renal artery, especially at depths of >4 mm.^8–10^

The average depth of radiofrequency-RDN, however, was derived from preclinical pathological studies using histopathologic sections cut at 3- to 5-mm intervals,^6–8^ which might miss the most representative sections. Hence, we should revisit the lesion depth reached by radiofrequency. The aim of this study was to find the true lesion depth per radiofrequency ablation in renal arteries at low- to high-output powers under fine sectioning (at 500-μm intervals). In addition, because fine sectioning enables linkage between biophysical parameters during ablation and lesion depth in a 1-to-1 manner, we investigated whether the biophysical parameters themselves could be predictors of lesion depth.

## Methods

The data, analytic methods, and study materials will not be made available to other researchers for purposes of reproducing the results or replicating the procedure because of intellectual property rights and product development confidentiality.

### Device Overview

**Figure 1.**
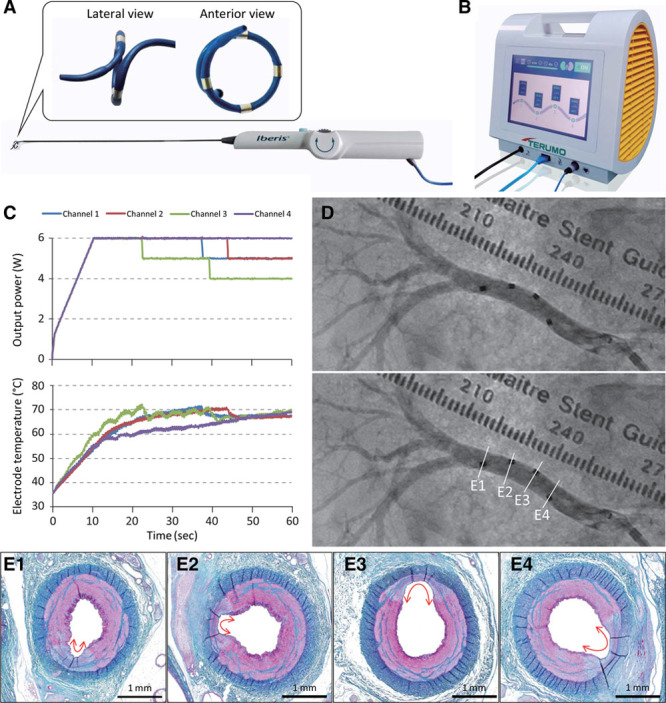
Overview of the IberisBloom renal denervation system. Helical multielectrode catheter (**A**) and the multichannel generator (**B**). **C**, Representative time courses of output power and electrode temperature of 4 channels at 6 W for 60 s. **D**, Representative angiographic images of the IberisBloom catheter deployed in a porcine renal artery. **E**, Histopathologic images of ablation lesions (red double arrows) correspond to each electrode position in (**D**) (**E1**–**E4**; elastica Masson trichrome stain).

The IberisBloom renal denervation system (Terumo Corporation, Tokyo, Japan) used in this study consists of a helical multielectrode catheter (Figure 1A) and its multichannel generator (Figure 1B). The catheter has 4 electrodes positioned to achieve helical treatment along the artery (Figure 1D and 1E). The generator delivers radiofrequency to selected electrodes simultaneously while monitoring electric impedance and electrode temperature. The IberisBloom system has an automatic control mechanism that decreases output power by 1 W in the channel when the corresponding electrode temperature is ≥70°C for 1 second to prevent excessive heating, potentially leading to unfavorable arterial damage (Figure 1C). For treatment with 2 W for 60 seconds (2 W/60 s), the prototype generator was used, which lacked the automatic control mechanism and simultaneous radiofrequency delivery to multiple electrodes. However, no electrode temperature reached 70°C during the treatment under the 2 W/60 s condition.

### Animals

All animal procedures were performed at the Evaluation Center at Terumo Corporation (Kanagawa, Japan) after approval of the Institutional Animal Care and Use Committee. The ablation catheter was applied bilaterally or unilaterally to 14 renal arteries in 9 female, crossbred (Landrace and Large White) pigs (57.9–66.3 kg). The ablation conditions were divided into 5 ablation settings: 2 W/60 s, 4 W/60 s, 6 W/60 s, 9 W/60 s, and 6 W/120 s (Table 1). All animals received aspirin (325 mg PO) daily from 3 days before the RDN procedure until the scheduled necropsy. Anticoagulation during the catheterization was achieved with intravenous heparin to maintain the activated clotting time at ≥250 seconds. A 7F introducer sheath was placed by percutaneous cannulation of the right femoral artery, and a 7F guide catheter was advanced to approach the renal arteries. Intra-arterial nitroglycerin (200 μg) was administered before angiography. After quantitative vessel analysis, renal arteries with diameters of 3 to 8 mm were ablated with the IberisBloom system (Figure 1). The length of the ablation segment was intentionally limited to 40 mm from the renal ostium and only one of several branch renal arteries. This method was necessary to avoid nonvertical dissection in peripheral branch arteries and fusion of the ablation lesions in branch arteries. The number of attempted ablation sites was recorded, and each site was labeled for matching with ablation lesions identified by histopathologic evaluation (Table 1).

**Table 1.**
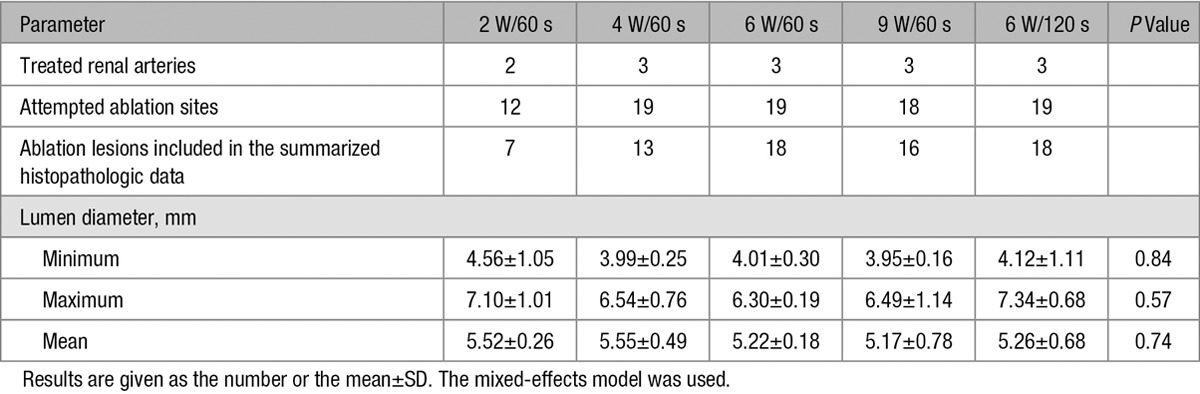
Sample Size and Lumen Diameters by Quantitative Vessel Analysis at Various Ablation Settings

At day 7, all animals underwent follow-up angiography after nitroglycerin administration (200 µg intra-arterial) and were then euthanized by exsanguination under general anesthesia. Renal arteries were perfusion-fixed at 100 mm Hg with 10% neutral-buffered formalin. Renal arteries with surrounding tissues and kidneys were harvested and immersed in formalin.

### Tissue Dissection and Paraffin Embedding

**Figure 2.**
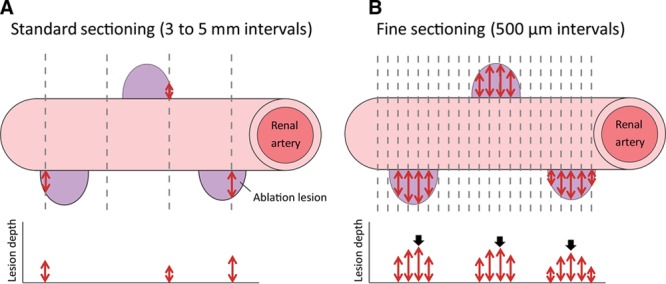
Standard (**A**) and fine (**B**) sectioning methods for histological evaluation. **A**, With the standard method, because the histological section does not always capture the center of the ablation lesion (purple), the average measured lesion depth (red double arrows) was theoretically less than that of the true lesion depth. **B**, With the fine sectioning method, several sections were produced from 1 lesion, and the lesion depth was measured for all sections. The maximum value (black arrows) per lesion was used to calculate the averaged maximum lesion depth at each ablation setting.

To avoid underestimating lesion depth, which is inevitable when using a standard sectioning method (Figure 2A), we adopted a fine sectioning method instead (Figure 2B). Each treated renal artery was sequentially cut from the ostium of the renal artery distally at 8-mm intervals, followed by delipidation, dehydration, and paraffin embedding. Each paraffin block was serially sectioned at 500-μm intervals and stained with hematoxylin-eosin and elastica Masson trichrome, resulting in 2470 sections (176±46 sections per artery).

### Histological Assessment

Treatment effects on the renal arteries and surrounding tissues were assessed on all produced sections under a light microscope with direct measurement and graded semiquantitatively on a scale of 0–4.^11^ The lesion depth was defined as the distance from the arterial lumen to that of the deepest damage, as measured with image analysis software (CellSens; Olympus, Tokyo, Japan). Endothelial loss was circumferentially evaluated as 0=no endothelial loss; 1=endothelial loss <25% of the vessel’s circumference; 2=endothelial loss of 25% to 50% of the vessel’s circumference; 3=endothelial loss of 51% to 75% of the vessel’s circumference; 4=endothelial loss of >75% of the vessel’s circumference. Medial change was evaluated separately by the depth and circumference of the involvement: 0=no medial change; grade 1=medial injury involving <25% of the medial depth/circumference; grade 2= medial injury 25% to 50% of the medial depth/circumference; grade 3=medial injury of 51% to 75% of the medial depth/circumference; grade 4=medial injury of >75% of the medial depth/circumference. Medial thinning was also evaluated according to the thickness of the media at the site of the damaged/unaffected media thickness of <0.5. Injury to nerves, arterioles, and fat were assessed as 0=none; 1=minimal; 2=mild; 3=moderate; 4=severe.

Based on the serial observation of the 500-μm interval sections, each ablation lesion created by radiofrequency delivered from a single electrode was identified. Each identified ablation lesion was matched with the corresponding attempted ablation site identified during angiography on day 0. The maximum depth and score were used to represent the score of each ablation lesion because the maximum value was considered to reflect the ablation effect more precisely than the mean value.^12,13^ In some cases, histological sections corresponding to the center of the ablation lesions were not obtained because the blade used for the histological dissection sometimes fell on the center unintentionally. Including histopathologic data from such deficient ablation lesions theoretically would lead to underestimating the actual maximum effects of the treatment. Thus, the data from the deficient ablation lesions were not included in the summarized data (Table 1).

### Biophysical Parameters

The generator automatically measured the following biophysical parameters in real time during delivery of radiofrequency: output power, impedance, and electrode temperature. The terminal output power was defined as the output power at a set time (ie, 60 or 120 seconds). Absolute impedance reduction (Ω) was calculated as the baseline (ie, 0 seconds) impedance minus the terminal impedance at a set time. Relative impedance reduction (%) was calculated as 100×absolute impedance reduction/baseline impedance. Peak electrode temperature was defined as the highest temperature during radiofrequency delivery. These parameters were documented per attempted ablation site.

### Statistical Analysis

The differences in the lumen diameters among the ablation settings were tested using a mixed-effects model with an animal as a random effect. For all histopathologic and biophysical parameters except for medial thinning, the linear trend against watt and the comparison between the 2 time settings (60 and 120 seconds) were analyzed using a mixed-effects model with a vessel within an animal as a random effect. For medial thinning, the linear trend against watt and the comparison between the 2 time settings were analyzed using a generalized mixed-effects model with a logit link function and with an animal as a random effect. The relationship between X (impedance reduction, peak electrode temperature, or maximum lesion depth) and Y (maximum lesion depth or nerve injury score) was evaluated by regression parameters using a mixed-effects model with Y as a response variable, with X as a fixed effect, and with a vessel within an animal as a random effect. Pearson correlation coefficients were also calculated between X and Y. A value of *P*<0.05 was considered to indicate statistical significance. All analyses were conducted with SAS software (version 9.4; SAS Institute Inc, Cary, NC).

## Results

All ablations were completed for the set time (60 or 120 seconds) without an abrupt impedance rise (which would indicate steam pops in the heated tissue or char formation on the electrodes). All treated animals survived until necropsy without any abnormalities caused by the RDN, as determined by daily clinical observation and then necropsy. There were no significant differences in the diameters of the treated renal arteries among the 5 ablation settings (Table 1). No angiographic abnormalities (eg, dissection, perforation, filling defects in the treated renal arteries, disruption of downstream flow) were found immediately after RDN or at 7 days.

**Figure 3.**
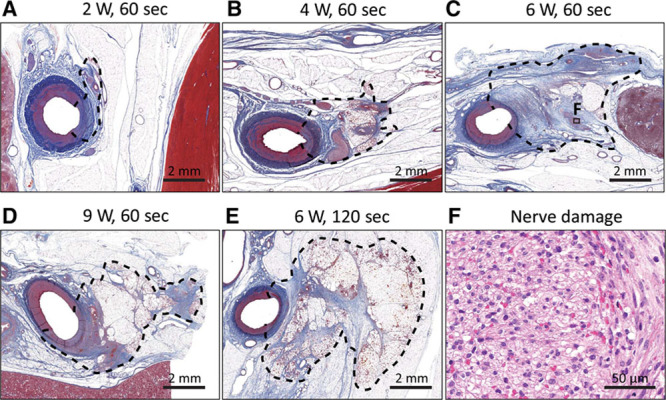
**A**–**E**, Representative histopathologic images of ablation lesions (dotted line) at each ablation setting (elastica Masson trichrome staining). **F**, Representative magnified image of a damaged nerve (boxed area, **C**). Note the marked vacuolization, formation of digestion chambers, pyknotic nuclei, infiltration of inflammatory cells, hemorrhage, and mild fibrosis in the perineurum and endoneurium (hematoxylin and eosin staining).

**Figure 4.**
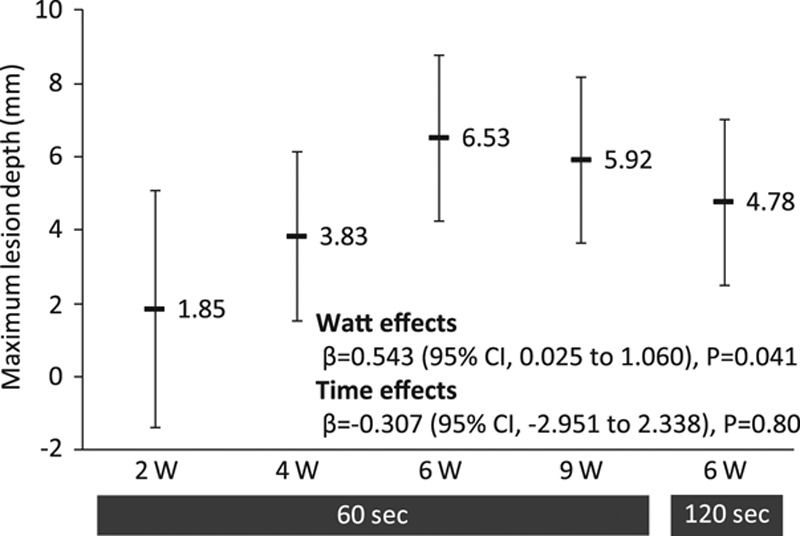
Maximum lesion depth averaged among multiple lesions at each ablation setting. The estimated mean values with 95% CI and the regression coefficients (β) with their *P* values are shown in the plots. The mixed-effects model was used. CI indicates confidence interval.

Histopathologic results are shown in Figures 3 and 4 and Table 2. The average maximum lesion depth increased in an output power-dependent manner at 2 to 6 W and peaked at 6.53 (95% confidence interval, 4.27–8.78) mm in the 6 W/60 s condition. It was not augmented by greater output power (9 W) or longer duration (120 seconds). The treated renal arterial lumen was almost completely re-endothelialized after ablation at all of the settings, as evidenced by low endothelial loss scores. Medial changes were transmural at all ablation settings. There was a statistically significant tendency toward higher scores for medial thinning, medial circumferential changes, nerve injury, arteriole injury, and fat injury at 6 and 9 W.

**Table 2.**
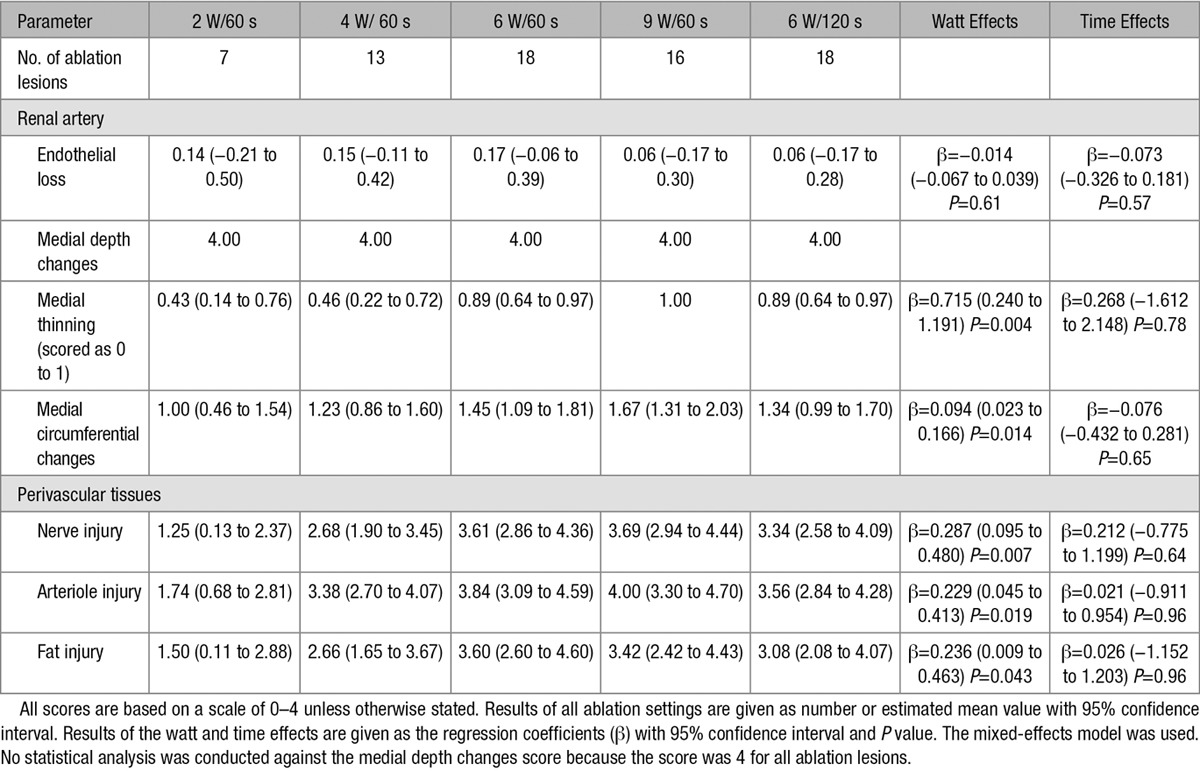
Histopathologic Semiquantitative Scores at Various Ablation Settings

The recorded biophysical parameters are summarized in Table 3. An automatic decrease in the output power in the IberisBloom system was activated more frequently in the greater output power settings, as shown in the discrepancy between the set output power and the terminal one. There was an increased tendency toward impedance reduction at 2 to 6 W and no substantial differences between 6 and 9 W. The peak electrode temperature increased consistently at 2 to 9 W.

**Table 3.**
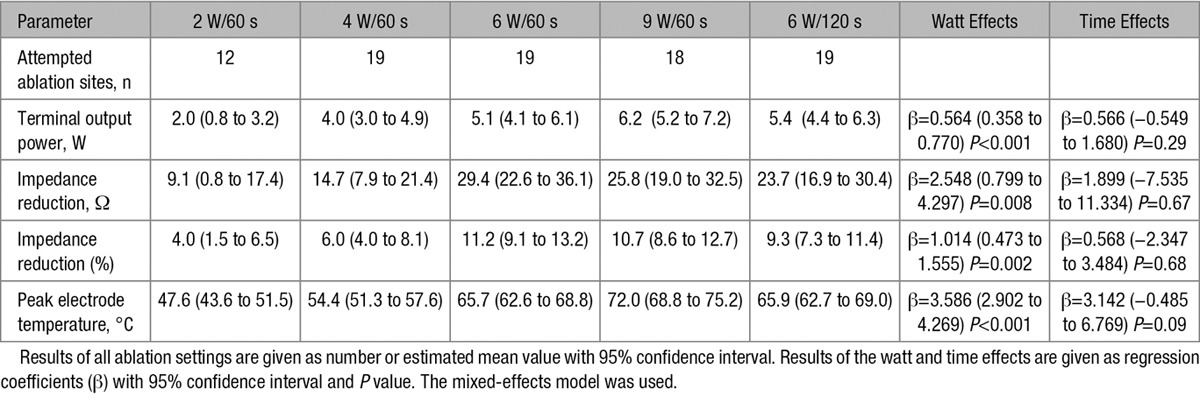
Biophysical Parameters at Various Ablation Settings

**Figure 5.**
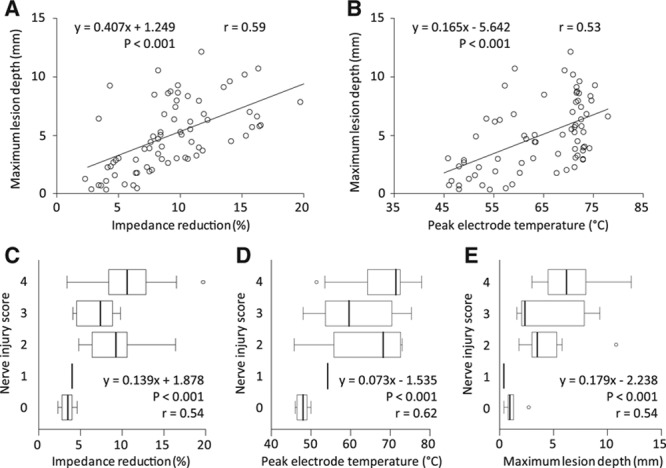
Scatter plots for maximum lesion depth and impedance reduction (**A**) and peak electrode temperature (**B**). Box plots for nerve injury scores and impedance reduction (**C**), peak electrode temperature (**D**), and maximum lesion depth (**E**). The regression equations, their *P* values, and Pearson correlation coefficients (*r*) are shown in the plots. The mixed-effects model was used except when calculating Pearson coefficients.

The maximum lesion depth for each ablation lesion was positively correlated with impedance reduction and the peak electrode temperature (Figure 5A and 5B), as was the nerve injury score (Figure 5C and 5D). The nerve injury score was also positively correlated with the maximum lesion depth (Figure 5E).

## Discussion

Using the fine sectioning (500-μm intervals) method (Figure 2B) for radiofrequency-RDN, we showed that the lesion depth from the arterial lumen was 6.5 mm under the 6 W/60 s condition (Figure 2A), which was deeper than that for standard sectioning (3- to 5-mm intervals).^6–8^ The lesion depth and radiofrequency-induced histopathologic changes were augmented in an output power-dependent manner at 2 to 6 W/60 s. No further augmentation was found at greater output power (9 W/60 s) or longer duration (6 W/120 s). In addition, we uniquely showed that the impedance reduction and peak electrode temperature during ablation were closely associated with the lesion depth of radiofrequency-RDN, suggesting that these biophysical parameters could be predictors of the lesion depth during radiofrequency-RDN.

In human renal arteries, a substantial number (28%–41%) of renal sympathetic nerves are located out of reach of the standard lesion depth by radiofrequency-RDN (3–4 mm),^5^ as deduced from earlier animal studies.^6–8^ There is concern that the depth of 3- to 4-mm depth achieved by radiofrequency was not sufficient for effective RDN, which might have led to the failure of the Symplicity HTN-3 trial.^3^ However, the true depth by radiofrequency had not been investigated in a sophisticated manner. Our results indicated that the maximum lesion depth was 6.5 mm at 6 W/60 s (the clinical setting of the IberisBloom system), which reached the depth containing the most renal sympathetic nerves (88% of the nerves are located at a depth of ≤6 mm).^5^ Moreover, a depth >6 mm may not be unique to our radiofrequency system (IberisBloom). It may be common in radiofrequency technology because other radiofrequency systems also showed ablation at a >5-mm depth in recent histopathologic animal studies.^13,14^ Therefore, the depth achieved by radiofrequency should not be the fundamental limitation for RDN in humans.

Although recent histopathologic studies suggest that radiofrequency-RDN is technologically able to ablate tissues at a >5-mm depth,^13,14^ there is a clear difference between those studies and ours. The earlier studies evaluated the maximum depth per artery, whereas we evaluated the maximum depth per lesion. Although the maximum depth per artery may not characterize depths of multiple lesions in an artery, the maximum depth per lesion is more important for effective circumferential RDN. Currently, ablation in all 4 anatomic quadrants (superior, inferior, anterior, posterior) is recommended by expert consensus^15^ based on the clinical finding of pronounced blood pressure reduction after 4-quadrant ablation.^16^ As renal sympathetic nerves are distributed in all 4 quadrants,^5^ the maximum depth per lesion is a better parameter for achieving circumferential RDN.

Theoretically, a lesion depth increases with time during an initial phase of radiofrequency delivery and then plateaus at thermal equilibrium,^17^ which has been demonstrated in harvested canine cardiac muscle.^18^ Because deeper lesions were not observed at a prolonged radiofrequency delivery duration of 120 seconds, we showed that the lesion depth plateaued at 6 W/60 s, suggesting the appropriate duration of radiofrequency-RDN as 60 seconds.

The relationship between biophysical parameters (impedance, electrode temperature) and the lesion depth created by radiofrequency has not been elucidated for RDN although there are some articles on cardiac ablation to treat arrhythmia.^19,20^ In this study, we showed that impedance reduction and the peak electrode temperature during ablation were closely associated with the lesion depth achieved by radiofrequency-RDN, which was in line with radiofrequency ablation in canine cardiac muscle.^19,20^ According to a linear regression formula, radiofrequency injury reaching 6 mm in depth is expected when the impedance reduction is ≥11.7% or the peak electrode temperature is ≥70.5°C. Considering the current major limitation that no definitive procedural success end point has been established for RDN,^15,21^ it is possible that the electrode temperature and impedance reduction could provide prompt feedback during radiofrequency-RDN to predict procedural success.

The failure of the SYMPLICITY HTN-3 trial cast doubt on the antihypertensive effects of radiofrequency-RDN.^3^ Considering the drawbacks in the trial design pointed out by post hoc analyses,^16^ new blinded and sham-controlled trials have been initiated: the SPYRAL HTN-OFF MED (Global Clinical Study of Renal Denervation With the Symplicity Spyral™ Multi-Electrode Renal Denervation System in Patients With Uncontrolled Hypertension in the Absence of Antihypertensive Medications) and SPYRAL HTN-ON MED trials (Global Clinical Study of Renal Denervation With the Symplicity Spyral™ Multi-Electrode Renal Denervation System in Patients With Uncontrolled Hypertension on Standard Medical Therapy).^22^ These trials were designed to exclude confounding factors associated with medication, patient selection, and RDN procedures to reprove the concept that radiofrequency-RDN reduces blood pressure in hypertensive patients. The results of the SPYRAL HTN-OFF MED trial recently showed that radiofrequency-RDN lowered blood pressure in patients with mild to moderate hypertension in the absence of antihypertensive medications while the blood pressure did not decrease in the sham-control arm.^23^ Although the efficacy of radiofrequency-RDN has not been established yet because of the small sample size of the trial, the trial demonstrated biological proof of principle that renal denervation lowers blood pressure in patients with untreated hypertension. Moreover, these findings emphasize the importance of radiofrequency-RDN devices as well as procedural techniques because there were differences in both radiofrequency-RDN devices and procedural techniques between the SYMPLICITY HTN-3 and SPYRAL HTN-OFF MED trials. Our study contributes to the development of radiofrequency-RDN by adding basic evidence on the denervation mechanism and potential predictors of procedural success.

### Study Limitations

The study had some limitations. First, the renal arteries of the normotensive pigs used in this study might be different from those of hypertensive humans. However, normotensive pigs are currently the standard preclinical model used to assess RDN devices because of their anatomic and physiological similarity to humans.^6–8,11,13,14^ Second, indicators of renal sympathetic function were not assessed (eg, renal norepinephrine concentration, tyrosine hydroxylase expression). However, these parameters would not have an impact on the study conclusion because our focus was to reveal the radiofrequency lesion depth per lesion at various ablation settings. Third, the prototype generator was used at 2 W/60 s, whereas the IberisBloom generator was used at other ablation settings. However, the difference of the generator specification would have no substantial influence on the ablation performance because no electrode temperature of 2 W/60 s reached 70°C, at which temperature the IberisBloom generator activates an automatic control mechanism. Fourth, the numbers of animals and vessels were relatively small. However, the fine sectioning method that was conducted in the present study was expected to minimize the variability in the measured lesion depth. Furthermore, the end point of the present study was lesion depth. The lesion depth should be less affected by individual differences than renal norepinephrine, which was measured as the end point in earlier studies.^6,7^ Fifth, the ablation target was limited to only the proximal segment of one branch artery as described in the Methods section despite the recent attention to ablation in all suitable branch renal arteries. This method was necessary because we gave priority to revealing the accurate depth of each ablation lesion by radiofrequency-RDN. Thus, the mutual effect of radiofrequency-RDN on branch ablation was not investigated in the present study. Sixth, it was statistically inappropriate to use Pearson correlation coefficient (*r*) in Figure 5 because each ablation lesion was nested in the renal arteries and the swine. We used Pearson correlation coefficients as referential indexes.

### Conclusions

The lesion depth per lesion was 6.4 mm in the treated renal arteries with the radiofrequency-delivering RDN device at 6 W/60 s, which was clarified by using a fine sectioning (at 500-μm intervals) method. This finding suggests that most of the renal sympathetic nerves could be ablated by radiofrequency-RDN. The impedance reduction and peak electrode temperature during ablation were closely associated with the lesion depth, indicating that these biophysical parameters could provide prompt feedback during radiofrequency-RDN procedures in the clinical setting.

## Acknowledgments

We gratefully acknowledge the contributions of Sept. Sapie Co, Ltd to the histological processing. We thank Nancy Schatken, BS, MT(ASCP), from Edanz Group, for editing a draft of this manuscript.

## Sources of Funding

This study was supported by Terumo Corporation, Tokyo, Japan.

## Disclosures

A. Sakaoka, H. Terao, Dr Nakamura, and H. Hagiwara are employees of Terumo Corporation. Dr Sakakura has received speaking honoraria from Abbott Vascular, Boston Scientific, Medtronic Cardiovascular, Terumo, OrbusNeich, and NIPRO. He has also served as a consultant for Abbott Vascular and Boston Scientific. The other authors report no conflicts.
